# *RP11-362K2.2:RP11-767I20.1* Genetic Variation Is Associated with Post-Reperfusion Therapy Parenchymal Hematoma. A GWAS Meta-Analysis

**DOI:** 10.3390/jcm10143137

**Published:** 2021-07-16

**Authors:** Elena Muiño, Jara Cárcel-Márquez, Caty Carrera, Laia Llucià-Carol, Cristina Gallego-Fabrega, Natalia Cullell, Miquel Lledós, José Castillo, Tomás Sobrino, Francisco Campos, Emilio Rodríguez-Castro, Mònica Millán, Lucía Muñoz-Narbona, Alejandro Bustamante, Elena López-Cancio, Marc Ribó, José Álvarez-Sabín, Jordi Jiménez-Conde, Jaume Roquer, Eva Giralt-Steinhauer, Carolina Soriano-Tárraga, Cristófol Vives-Bauza, Rosa Díaz Navarro, Silvia Tur, Victor Obach, Juan F. Arenillas, Tomás Segura, Gemma Serrano-Heras, Joan Martí-Fàbregas, Raquel Delgado-Mederos, Pol Camps-Renom, Luis Prats-Sánchez, Daniel Guisado, Marina Guasch, Rebeca Marin, Alejandro Martínez-Domeño, Maria del Mar Freijo-Guerrero, Francisco Moniche, Juan Antonio Cabezas, Mar Castellanos, Jerzy Krupinsky, Daniel Strbian, Turgut Tatlisumak, Vincent Thijs, Robin Lemmens, Agnieszka Slowik, Joanna Pera, Laura Heitsch, Laura Ibañez, Carlos Cruchaga, Rajat Dhar, Jin-Moo Lee, Joan Montaner, Israel Fernández-Cadenas, on behalf of International Stroke Genetic Consortium, the Spanish Stroke Genetic Consortium

**Affiliations:** 1 Stroke Pharmacogenomics and Genetics Group, Institut de Recerca de l’Hospital de la Santa Creu i Sant Pau, 08041 Barcelona, Spain; elena.muinho@gmail.com (E.M.); jara.carcel@gmail.com (J.C.-M.); laialluciacarol@gmail.com (L.L.-C.); cristina.gallego.fabrega@gmail.com (C.G.-F.); natalia.cullell@gmail.com (N.C.); miquel.lledos@gmail.com (M.L.); 2 Neurovascular Research Laboratory, Vall d’Hebron Institut de Recerca, Universitat Autònoma de Barcelona, 08025 Barcelona, Spain; catycarrerav@gmail.com; 3 Department of Neurology, Hospital de la Santa Creu i Sant Pau, IIB-Sant Pau, 08025 Barcelona, Spain; jmarti@santpau.cat (J.M.-F.); rdelgado@santpau.cat (R.D.-M.); pcamps@santpau.cat (P.C.-R.); LPratsS@santpau.cat (L.P.-S.); DGuisado@santpau.cat (D.G.); MGuasch@santpau.cat (M.G.); rmarin@santpau.cat (R.M.); amartinezd@santpau.cat (A.M.-D.); 4 Stroke Pharmacogenomics and Genetics, Fundació MútuaTerrassa per la Docència i la Recerca, 08221 Terrassa, Spain; 5 Clinical Neurosciences Research Laboratories, Health Research Institute of Santiago de Compostela (IDIS), 15706 Santiago de Compostela, Spain; jose.castillo.sanchez@sergas.es (J.C.); Tomas.Sobrino.Moreiras@sergas.es (T.S.); francisco.campos.perez@sergas.es (F.C.); 6 Department of Neurology, Hospital Clínico Universitario de Santiago, 15706 Santiago de Compostela, Spain; emiliorcastro@gmail.com; 7 Department of Neuroscience, Hospital Germans Trias i Pujol, 08025 Badalona, Spain; mmillan.germanstrias@gencat.cat (M.M.); luciamunozn@gmail.com (L.M.-N.); alebustamanterangel@gmail.com (A.B.); 8 Stroke Unit, Hospital Universitario Central de Asturias, 33011 Oviedo, Spain; elenacancio@gmail.com; 9 Stroke Unit, Hospital Universitario Valle de Hebrón, 08025 Barcelona, Spain; marcriboj@hotmail.com; 10 Department of Neurology, Hospital Universitario Valle de Hebrón, Universidad Autónoma de Barcelona, 08025 Barcelona, Spain; josalvar@vhebron.net; 11 Department of Neurology, Neurovascular Research Group, Instituto de Investigaciones Médicas Hospital del Mar-Hospital del Mar, 08025 Barcelona, Spain; jjimenez@imim.es (J.J.-C.); jroquer@hospitaldelmar.cat (J.R.); egiralt@imim.es (E.G.-S.); csoriano@imim.es (C.S.-T.); 12 Neurobiology Laboratory, Instituto de Investigación Sanitaria de Palma, 07120 Mallorca, Spain; cristofol.vives@ssib.es; 13 Department of Neurology, Hospital Universitari Son Espases, 07120 Mallorca, Spain; rosam.diaz@ssib.es (R.D.N.); silvia.tur@ssib.es (S.T.); 14 Department of Neurology, Hospital Clínic i Provincial de Barcelona, 08025 Barcelona, Spain; VOBACH@clinic.cat; 15 Department of Neurology, Hospital Clínico Universitario, University of Valladolid, 47003 Valladolid, Spain; juanfarenillas@gmail.com; 16 Department of Neurology, Complejo Hospitalario Universitario de Albacete, 02006 Albacete, Spain; tseguram@gmail.com; 17 Experimental Research Unit, Complejo Hospitalario Universitario de Albacete, 02006 Albacete, Spain; gemmas@sescam.jccm.es; 18 Neurovascular Unit, Biocruces Bizkaia Health Research Institute, 48903 Bilbao, Spain; marimar.freijoguerrero@osakidetza.eus; 19 Department of Neurology, Virgen del Rocío, Instituto de Biomedicina de Sevilla, 41013 Seville, Spain; pmoniche@gmail.com (F.M.); juancaro.jacr@gmail.com (J.A.C.); joan.montaner@vhir.org (J.M.); 20 Department of Neurology, Complejo Hospitalario Universitario A Coruña, 15006 A Coruña, Spain; Maria.del.Mar.Castellanos.Rodrigo@sergas.es; 21 School of Healthcare Science, Manchester Metropolitan University, Manchester M15 6BH, UK; jkrupinski@mutuaterrassa.es; 22 Neurology Unit, Hospital Universitari Mútua Terrassa, 08221 Terrassa, Spain; 23 Department of Neurology, Helsinki University Hospital, FI-00029 Helsinki, Finland; daniel.strbian@hus.fi; 24 Department of Clinical Neuroscience, Institute of Neurosciences and Physiology, Sahlgrenska Academy at University of Gothenburg, 41345 Gothenburg, Sweden; turgut.tatlisumak@neuro.gu.se; 25 Department of Neurology, Sahlgrenska University Hospital, 41345 Gothenburg, Sweden; 26 Stroke Division, Florey Institute of Neuroscience and Mental Health, University of Melbourne, Heidelberg VIC 3072, Australia; vincent.thijs@austin.org.au; 27 Department of Neurology, Austin Health, Heidelberg VIC 3072, Australia; 28 Department of Neurology, University Hospitals Leuven, Campus Gasthuisberg, 3000 Leuven, Belgium; robin.lemmens@uzleuven.be; 29 Department of Neurology, Jagiellonian University Medical College, 31-007 Kraków, Poland; slowik@neuro.cm-uj.krakow.pl (A.S.); pera@su.krakow.pl (J.P.); 30 Division of Emergency Medicine, Washington University School of Medicine, St. Louis, MO 63110-1010, USA; lheitsch@wustl.edu; 31 Department of Neurology, Washington University School of Medicine, St. Louis, MO 63110-1010, USA; dharr@wustl.edu (R.D.); leejm@wustl.edu (J.-M.L.); 32 Department of Psychiatry, Washington University School of Medicine, St. Louis, MO 63110-1010, USA; ibanezl@wustl.edu (L.I.); cruchagac@wustl.edu (C.C.)

**Keywords:** hemorrhagic transformation, parenchymal hematoma, GWAS, single nucleotide variants

## Abstract

Stroke is one of the most common causes of death and disability. Reperfusion therapies are the only treatment available during the acute phase of stroke. Due to recent clinical trials, these therapies may increase their frequency of use by extending the time-window administration, which may lead to an increase in complications such as hemorrhagic transformation, with parenchymal hematoma (PH) being the more severe subtype, associated with higher mortality and disability rates. Our aim was to find genetic risk factors associated with PH, as that could provide molecular targets/pathways for their prevention/treatment and study its genetic correlations to find traits sharing genetic background. We performed a GWAS and meta-analysis, following standard quality controls and association analysis (fastGWAS), adjusting age, NIHSS, and principal components. FUMA was used to annotate, prioritize, visualize, and interpret the meta-analysis results. The total number of patients in the meta-analysis was 2034 (216 cases and 1818 controls). We found rs79770152 having a genome-wide significant association (beta 0.09, *p*-value 3.90 × 10^−8^) located in the *RP11-362K2.2:RP11-767I20.1* gene and a suggestive variant (rs13297983: beta 0.07, *p*-value 6.10 × 10^−8^) located in *PCSK5* associated with PH occurrence. The genetic correlation showed a shared genetic background of PH with Alzheimer’s disease and white matter hyperintensities. In addition, genes containing the ten most significant associations have been related to aggregated amyloid-β, tau protein, white matter microstructure, inflammation, and matrix metalloproteinases.

## 1. Introduction

Stroke is the second most common cause of death worldwide, and the third most common cause of disability [1]. For ischemic strokes, the only treatments available during the acute phase are the reperfusion therapies such as thrombolysis and mechanical thrombectomy.

Ischemic strokes may present hemorrhagic transformation (HT). This may be early, associated with reperfusion of the occluded vessel; or late, which is thought to be related to increased permeability and blood flow [2].

HT is a well-recognized complication following reperfusion therapies. HT could be classified, according to the European Cooperative Acute Stroke Study (ECASS) criteria, into petechial infarction without space-occupying effect (HI) and hematoma/coagulum with mass effect (PH) [2].

HT may result in neurological deterioration [3], and the presence of a PH independently predicts early and late mortality, with a hazard ratio of late mortality of 7.9, with a 95% confidence interval (CI) of 2.9–21.4 [4]. Nevertheless, petechial changes may indicate that reperfusion occurred when the ischemic tissue was still at least partially viable.

Patients exhibiting an early HI did not have a higher risk of neurological deterioration compared with patients without hemorrhagic transformation. Among patients treated with rtPA, HI was even loosely associated with early improvement. Overall, three-month mortality and disability were also not influenced by HI [2].

The percentage of HT in studies of stroke patients varies from 6.4% to 43% [3], and the use of reperfusion therapies has favored the increase in this incidence. Moreover, clinical trials such as WAKE-UP [5], DAWN [6], or DEFUSE 3 [7] will allow a major use of these therapies, extending the time-window administration, which may lead to an increase in HT. It is therefore of utmost importance to identify those patients at higher risk of suffering a PH, as this is the subtype of HT that causes the highest morbidity and mortality [2,4].

There is a genetic predisposition for HTs following intravenous thrombolysis (IVT). This genetic contribution has been explored through candidate genes [8,9] or more recently through a Genome Wide Association Study (GWAS), carried out by our own group [10]. In this last study, we found that single nucleotide variants (SNVs) in the *ZBTB46* gene were associated with PH in patients who underwent IVT [10]. For this purpose, we studied the extreme phenotype, patients with PH vs. patients without HT, excluding patients with petechial infarction (HI) subtype.

We decided to carry out a new analysis by including in the control group those patients who had a HI, to ensure that the findings achieved are exclusively attributed to the PH subtype due to reperfusion therapies, including patients that underwent mechanical thrombectomy or intra-arterial fibrinolysis, increasing our sample size, and with it, our statistical power.

Currently, articles using GWAS to understand different diseases are complemented by the study of genetic correlations with other traits to find common genetic architecture [11]. Knowing which traits share a genetic correlation allows a better understanding of diseases and the realization of further studies to find variants associated with them by increasing its statistical power, such as multitrait analysis of GWAS (MTAG). As example, the article performing a MTAG of small vessel occlusion strokes and intracerebral hemorrhage, due to these traits sharing a genetic background, allows us to find new loci associated with these diseases [12].

In the article we mentioned above, published by our group, we found that PH shared a genetic background with deep intracerebral hemorrhage (ICH), lobar ICH, and white matter hyperintensities (WMH) [10]. After Bonferroni correction, only lobar ICH remained significantly correlated.

Therefore, the aim of our study was to find genetic risk factors associated exclusively with PH, including patients with different reperfusion treatments. PH occurrence is still an important problem in the reperfusion strategy for stroke patients. Hence the importance of finding molecules that could be used as biomarkers to guide the therapeutic decision or potential therapeutic targets to prevent the appearance of this life-threatening complication. We also wanted to assess whether the same genetic correlations found in our previous paper were still found and whether we could find any new ones.

In this work we found a genome-wide significant locus associated with PH, regardless of the reperfusion treatment performed. Moreover, we found that there is a genetic correlation of PH with Alzheimer’s disease and white matter hyperintensities (WMH). In fact, the study of nominally significant genomic loci in the meta-analysis has shown that pathways related to aggregated amyloid-β, tau protein, and inflammatory pathways could be related to PH occurrence.

## 2. Materials and Methods

This is an observational case-control study, conducted in a discovery and replication cohort, with subsequent meta-analysis of both results, in order to find SNVs associated with PH.

### 2.1. Subjects

#### 2.1.1. Discovery Cohort

The participants included in the discovery cohort were part of the Genetic Study in Ischemic Stroke Patients treated with recombinant tissue plasminogen activator (r-tPA) (GenoTPA) [9], Genetic contribution to Functional Outcome and Disability after Stroke (GODS) [13], the Genotyping Recurrence Risk of Stroke (GRECOS) [14], and Genetics of Early Neurological Instability After Ischemic Stroke (GENISIS) [15] studies. These studies have, in common, the recruitment of patients with ischemic stroke between 2002 and 2020.

From these four studies, (*n* = 4667), 161 cases (patients with PH after reperfusion therapy) and 1236 controls (patients without PH after reperfusion therapy) fulfilled the inclusion and exclusion criteria, incorporated in a total of 8 batches (Table 1). All of the subjects of the discovery cohort had a Spanish origin.

#### 2.1.2. Replication Cohort

The participants included in the replication cohort were part of the Genetic Study in Ischemic Stroke Patients treated with tPA (GenoTPA) [9], BAse de Datos de ICtus del hospital del MAR (BASICMAR) (Stroke database of the Hospital del Mar) [16], Leuven Stroke Genetics Study (LSGS) [17], Helsinki 2000 Ischemic Stroke Genetics Study, and Genetics of Early Neurological Instability After Ischemic Stroke (GENISIS) [15] studies.

From these five studies, the imputed genotype was available from a total of 1064 patients, 112 cases and 913 controls, incorporated in a total of 7 batches (Table 2).

For a detailed description of the different studies included in the discovery and replication cohorts see Supplemental Methods.

#### 2.1.3. Variables

Detailed clinical-epidemiological data was collected from each patient, including age, sex, vascular risk factors such as hypertension, diabetes mellitus (DM), dyslipidemia (DLP), smoking habits, history of atrial fibrillation (AF), physical examination including stroke severity assessed with the National Institutes of Health Stroke Scale (NIHSS) at initial evaluation and the modified Rankin Score (mRS) prior to stroke, systolic (SBP) and diastolic blood pressure (DBP), initial glycaemia, TOAST classification, or treatment decisions. In Supplemental Methods, there is detailed information about variable definition.

CT scans were obtained prior to reperfusion procedure (baseline), and 24 h after, or whenever a neurological deterioration detected by the clinician was observed, to assess the presence of HT and its degree. All brain images were reviewed by a radiologist or neuro-radiologist.

HT was classified, according to the ECASS criteria, into petechial infarction without space-occupying effect (HI) with two subtypes, HI1 (small petechiae) and HI2 (more confluent petechiae); and hematoma/coagulum with mass effect (PH) divided into PH1 when affecting ≤30% of the infarct bed with mild mass effect and PH2, when affecting >30% of the infarct bed with significant mass effect or remote hemorrhage [2].

As the aim of our study was to find SNV associated with the risk of PH (PH1 and PH2) after reperfusion treatment, patients without HT or with HI (HI1 and HI2) were chosen as controls, and patients with PH were chosen as cases. Remote hemorrhages were excluded from the study, as their etiology has not yet been clarified and the biological mechanisms underlying remote hemorrhages are probably different compared to the other HTs [18].

#### 2.1.4. Eligibility Criteria

For the association study, patients >18 years of age with an ischemic stroke that underwent reperfusion therapy (ITV, including mechanical thrombectomy or intra-arterial fibrinolysis as second intention), who presented with PH, were considered as cases. Controls were selected as patients >18 years with ischemic stroke that underwent reperfusion therapy, who did not present HT or who presented with HI.

Exclusion criteria: patients not receiving reperfusion therapy, who suffered a remote PH or unknown HT phenotype.

#### 2.1.5. Standard Protocol Approvals and Patient Consent

This study was approved by the local ethics committee of each participant and an informed consent document was signed by every patient or their relatives.

### 2.2. Genotyping

DNA samples were genotyped on commercial arrays from Illumina (San Diego, CA, USA) (Table 1 and Table 2).

#### 2.2.1. Quality Control

For detailed quality controls performed see Supplemental Methods.

Briefly, SNV missing in a large proportion of the subjects, non-biallelic SNV, ambiguous, monomorphic or duplicated SNV, or SNV that violates the Hardy–Weinberg (dis)equilibrium (HWE) law were deleted.

Individuals with high rates of genotype missingness, sex discrepancy or unknown sex, family members or duplicated samples, non-European individuals, and patients with outlier heterozygosity rates (*n* = 814) were removed.

After all these QCs, the total number of patients was 141 cases and 1003 controls in the discovery cohort. To ensure that there were no duplicate samples between the discovery and replication cohorts, patients with a pihat > 0.8 were removed from replication cohort. The number of patients with information for the covariates introduced in the analysis were 1139, 140 cases and 999 controls.

Finally, 895 patients (76 cases and 819 controls) passed the QC and had information for the covariates in the analysis, constituting the replication cohort.

Studies genotyped on the same platforms were combined in the discovery cohort. For the replication cohorts data were already imputed [10].

#### 2.2.2. Genome Build

All genomic coordinates are given in NCBI Build 37/UCSC hg19.

### 2.3. Imputation

Imputation was performed with the Michigan Imputation Server Pipeline using Minimac4, following their instructions (https://imputationserver.readthedocs.io/en/latest (accessed on 1 May 2021)). HRC r1.1 2016 (GRCh37/hg19) was the reference panel used, with European population and, for phasing, Eagle v2.4 was used.

After imputation, QC were performed. We removed SNV with r^2^ < 0.6 and MAF < 0.1%. After merging all cohorts, SNVs that were not present in at least 90% of the individuals were removed.

### 2.4. Genome-Wide Association Analysis and Meta-Analysis

**Figure 1:**
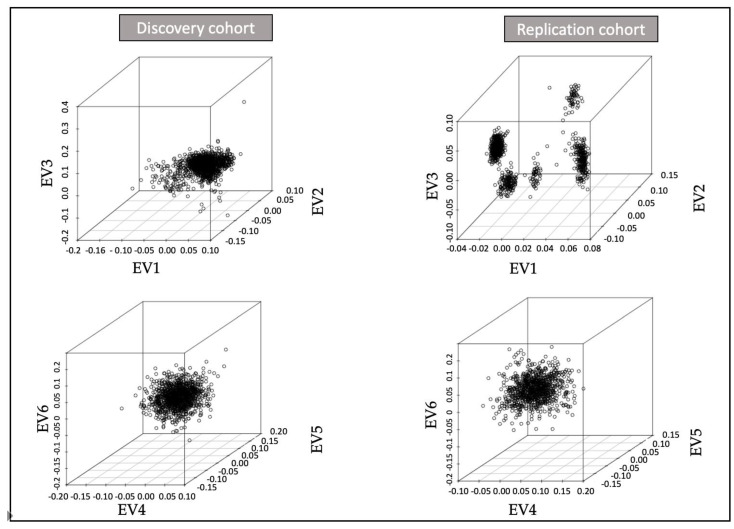
Principal component analysis (PCA) representation for discovery and replication cohorts. EV: eigenvector.

We performed a linear regression-based association analysis using fastGWAS [19]. Those SNV with minor allele count (MAC) < 6 were subsequently removed. For the discovery cohort, we adjusted for the first two principal components (PC) (Figure 1), age and the variables remaining significant in the multivariable logistic regression (*p*-value < 0.05) and that we had information on the replication cohort: NIHSS. For the replication cohort, the analysis was adjusted for the three first PC (Figure 1), and the same clinical variables as in the discovery analysis: age and NIHSS.

Due to the small sample size of the discovery cohort, in order to increase statistical power, we carried out a meta-analysis of the results of the discovery and replication cohort with the metal software (http://csg.sph.umich.edu/abecasis/metal (accessed on 5 May 2021)), weighted by the number of individuals contributing to each result [20]. Genomic control correction was applied to both input files and then to the meta-analysis results.

A *p*-value < 5 × 10^−8^ was considered genome-wide significant and a *p*-value < 1 × 10^−5^ a nominal genome-wide association.

### 2.5. Functional Annotation of Associated Variants

FUMA (Functional Mapping and Annotation of Genome-Wide Association Studies) was used to annotate, prioritize, visualize, and interpret the meta-analysis results (https://fuma.ctglab.nl (accessed on 6 May 2021)) [21]. This platform also permits the realization of an ANNOVAR enrichment test; MAGMA gene analysis, gene-set analysis and gene-property analysis; identification of expression quantitative trait loci (eQTL), chromatin interaction data, and mapping. It also provides information about the RegulomeBD score. This score, that provides information on the probability of affect binding and expression of target gene, goes from 1 (most likely) to 7 (least likely). As a reference panel, we used UKB release2b 10k European population.

To search for traits to which the genes closest to the most significant SNVs have been related, we used the GWAS Catalog (https://www.ebi.ac.uk/gwas (accessed on 6 May 2021)).

For finding gene ontology (GO) terms of the genes of interest, we performed a search in Ensembl (https://www.ensembl.org/index.html (accessed on 6 May 2021)).

### 2.6. Estimation of Genetic Correlations

We used GNOVA (GeNetic cOVariance Analyzer) to estimate genetic covariance and correlation between traits. For this estimation, GNOVA only requires the genetic information available in the summary statistics of the traits of interest.

We tested genetic correlation for traits that have been related with HT: any ischemic strokes (AIS, *n* = 440,328), large artery atherosclerosis strokes (LAS, *n* = 301,663), cardieombolic strokes (CES, *n* = 362,661), and small vessel occlusion strokes (SVO, *n* = 348,946) using MEGASTROKE European data [22], deep intracerebral hemorrhage (*n* = 2075) [23], lobar intracerebral hemorrhage (*n* = 1148) [23], white matter hyperintensities (WMH, *n* = 11,226) [24], Alzheimer’s disease (AD, *n* = 63,926) [11], total cholesterol (*n* = 94,595) [25], LDL (*n* = 94,595) [25], HDL (*n* = 94,595) [25], triglycerides (*n* = 94,595) [25], sistolic blood pressure (SBP, *n* = 757,601) [26], diastolic blood pressure (DBP, *n* = 757,601) [26], and diabetes mellitus 2 (DM2) (*n* = 69,033) [27].

### 2.7. Statistical Analyses

R version 3.6.3 and Bioconductor packages were used to perform the statistical analysis. To study whether there were significant differences (*p*-value < 0.05) between cases and controls in the discovery and replication cohorts, for quantitative variables with a normal distribution, we used *t*-test and a Mann–Whitney U for non-normal quantitative or ordinal variables. The Chi-square test was used for categorical variables.

Multivariable logistic regression was conducted following a forward stepwise approach to select clinical variables as covariates for the association study. First, univariable logistic regression was performed to study the association between the available variables and the occurrence of PH. Then, they were added to the multivariable logistic regression model according to their *p*-value, from the most significant to the least.

Variables with more than 10% missing values (less than 1030 observations) were not taken into account for the multivariate model (DLP, smoking habits, mRS, SBP, DBP, intra-arterial fibrinolysis, and mechanical thrombectomy), as the results of subsequent statistical analyses might be biased [28] and the analysis underpowered.

### 2.8. Data Availability

The data that supports the findings of this study is available from the corresponding author upon reasonable request.

## 3. Results

### 3.1. Descriptive Analysis of the Cohorts

#### 3.1.1. Discovery

A total of 1144 patients with an ischemic stroke, and who were treated with reperfusion treatment, met the inclusion criteria and passed the QC; a total of 1139, with 140 cases and 999 controls, had information for the covariates of the analysis. A total of 10,058,599 SNP passed QC and were evaluated.

There was a total of 141 cases with PH (12%) and 1003 controls (88%). Of these controls, 840 had no hemorrhagic transformation (84%) and 163 had HI (16%). Cases were 77 ± 12 years old (median ± interquartile range -IQR-), 52% were males, 13% (11/88) received intra-arterial fibrinolysis, and none received mechanical thrombectomy. Controls were 75 ± 16 years old (median ± IQR), 55% were males, 5% (28/620) received intra-arterial fibrinolysis, and 6% (17/286) mechanical thrombectomy. In the univariable analysis, the variables significantly associated with PH were a higher NIHSS, higher mean mRS (0.83 vs. 0.46 in controls), higher percentage of intra-arterial fibrinolysis, and lower percentage of strokes of atherothrombotic etiology. The detailed descriptive analysis can be found in Table 3.

The final sample for the analysis with information for all the covariates included in the association test was 1139 patients, with 140 cases and 999 controls.

In the multivariate analysis with age and the first two PCs, only NIHSS remains significant (*p*-value 5.36 × 10^−3^). Variables with a miss rate >10% or those that were not collected in the replication cohort were excluded from this analysis.

#### 3.1.2. Replication

A total of 895 patients with an ischemic stroke undergoing reperfusion treatment, met the inclusion criteria and passed the QC. A total of 7,224,265 SNP after QCs were evaluated.

There was a total of 76 cases with PH (8%) and 819 controls (92%). Cases were 76 ± 11 years old (median ± IQR) and 53% were males. Controls were 72 ± 17 years old (median ± IQR) and 52% were males. In the univariable analysis, the variables significantly associated with PH were a higher age, a higher proportion of AF and CES, and a higher NIHSS. The detailed descriptive analysis can be found in Table 4.

The final sample for the analysis with covariates was 895 patients, 76 cases and 819 controls.

### 3.2. GWAS

We did not observe any SNV that reached the GWAS significance threshold (*p*-value < 5 × 10^−8^) in the discovery analysis.

The Manhattan and quantile-quantile (QQ) plots, obtained from the discovery and replication cohorts association study, can be visualized in the supplementary Figures S1 and S2, respectively. We did not observe an overall inflation of *p*-values; genomic inflation factor λ was 1.007 in the discovery cohort and 0.999 in the replication.

### 3.3. Meta-Analysis

With the meta-analysis, we found a genomic locus with a significant genome-wide association (*p*-value <5 × 10^−8^). This genomic locus is constituted by 57 SNV in our meta-analysis (Supplementary Table S1). Its leading SNV is 12:59127963:A:G (rs79770152) and it is an intronic variant located in the RP11-362K2.2:RP11-767I20.1 gene, with a *p*-value of 3.90 × 10^−8^ (MAF: 0.09; Beta coefficient: 0.09, standard error (SE): 0.015).

In addition, a total of 28 genomic loci with nominal SNV were found (*p*-value < 1.00 × 10^−5^) (Supplementary Table S2). One of these loci contains a leading SNV that almost reaches statistical significance at genome-wide level, 9:78563802:G:T (rs13297983). It is an intronic variant located in the gene PCSK5 with a *p*-value of 6.10 × 10^−8^ (MAF: 0.07; Beta coefficient: 0.097, SE: 0.017).

**Figure 2:**
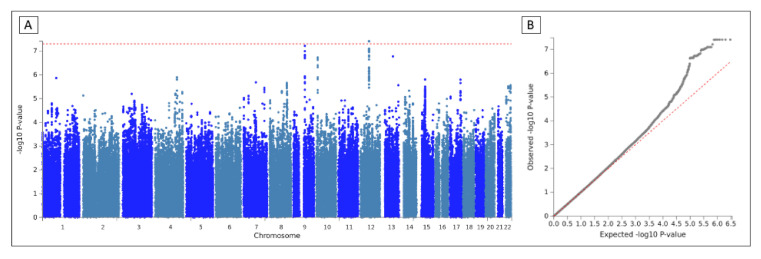
Manhattan and QQ plot of the meta-analysis. (**A**) Manhattan plot. SNVs were represented by dots and plotted based on their genome-wide association study *p*-values. Red line shows genome-wide significance (*p*-value < 5 × 10^−8^). (**B**) QQ plot of the *p*-values obtained after the association testing. The x-axis represents the expected −log_10_—*p*-value under the null hypothesis and lambda is the median of the resulting chi-squared test statistics divided by the expected median of the chi-squared distribution under the null hypothesis.

None of these two SNVs are eQTL or present chormatin interactions regarding the databases available in FUMA. Table 5 shows the description of the top ten genomic loci with the most significant SNV and Figure 2 the Manhattan plot.

One of the SNV belonging to one of this top ten genomic loci (17:72393744:A:G, rs4348170, *p*-value 1.60 × 10^−6^) has been associated in another GWAS with interleukin levels [28]. If we perform a GWAS Catalog search for the genes closest to the leading SNVs of these genomic loci, we find that variants of PCSK5 have been associated with diffuse plaques of aggregated amyloid-β peptide in the brain, measurement of tau protein in the form of paired helical filaments, apolipoproteina B, or LDL levels regarding the consumption of alcohol. KLF5 with neutrophil and monocyte count or lymphocyte percentage of leukocytes. TGFBR3 with multiple sclerosis and pulse pressure measurement. C15orf48 with urinary albumin to creatinine ratio, glomerular filtration rate, and albuminuria. RNA5SP448 with LDL and interleukin 12 measurement. SEMA3A with white matter microstructure measurement, cortical thickness, major depression, and alcohol dependence or DNA methylation. EIF3H with neurofibrillary tangles.

**Figure 3:**
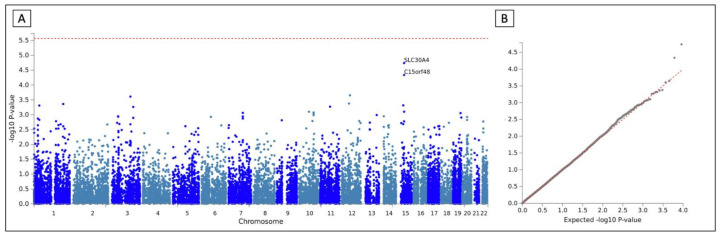
Manhattan and QQ plot of the gene-based meta-analysis. (**A**) Manhattan plot. Genes were represented by dots and plotted based on their *p*-values. Red line shows the considered significant *p*-value (*p* < 5 × 10^−8^). (**B**) QQ plot of the *p*-values obtained after the association testing. The x-axis represents the expected −log_10_—*p*-value under the null hypothesis and lambda is the median of the resulting chi-squared test statistics divided by the expected median of the chi-squared distribution under the null hypothesis.

Gene-based analysis performed with FUMA took into account a total of 18317 protein coding genes. Therefore, the significant *p*-value corrected for multiple comparisons was 2.73 × 10^−6^. None of the genes reached statistical significance. The most significant associations were SLC30A4 (*p*-value 1.82 × 10^−5^) and C15orf48 (*p*-value 4.58 × 10^−5^), both in chromosome 15 (Figure 3).

### 3.4. MAGMA Analysis and GO Terms

FUMA platform performs MAGMA gene-set analysis for curated gene sets and gene ontology (GO) terms obtained from MsigDB. The only significant association after adjusting for the Bonferroni method was the GO term (molecular function) myosin V binding (adjusted *p*-value 2.04 × 10^−3^), which definition is the interaction selectively and non-covalently with a class V myosin. Supplementary Table S3 shows the top ten of the most significant curated gene sets and GO terms.

The most relevant GO terms could be visualized on Table 5.

### 3.5. Genetic Correlations

Genetic correlation analysis detected a shared genetic background among PH presence and Alzheimer’ Disease and white matter hyperintensities (WMH) with a raw *p*-value < 0.05 (Table 6). None of the traits reached a significant *p*-value adjusted for multiple comparisons (*p*-value adjusted with Bonferroni method: 4.16 × 10^−3^).

## 4. Discussion

This is an observational case-control study in order to find genetic risk factors and biological mechanisms associated with brain parenchymal hemorrhagic transformation after reperfusion treatment in ischemic stroke.

In a previous work by our group, we explored which SNVs were associated with hemorrhagic transformation through a GWAS, analyzing extreme phenotypes: PH vs. non hemorrhagic transformation in patients undergoing only IVT [10]. This led to the finding that rs7648433, located in *ZBTB46* gene, was associated with this phenotype and it has been implicated in mechanisms such as shear stress and atherosclerosis in other studies.

In the current study, we analyzed patients undergoing IVT and including, additionally, patients with intra-arterial fibrinolysis or mechanical thrombectomy. We wanted to obtain more generalized results, as these therapies are widely used and their window time administration has recently been increased [5,6,7]. This longer time-window administration may lead to an increase of hemorrhagic complications, one of the major problems of these treperfusion therapies. Understanding why a patient may develop PH including patients underwent any type of reperfusion treatment may be of great interest, as this subtype is the one with the highest rates of morbi-mortality [2,4].

In addition, we have added other HT subtypes different from PH to the group of controls (HI). This strategy is interesting to find genetic risk factors associated exclusively to PH in contrast to our previous work [10], as we are avoiding any possible genetic risk factor that could be associated to both, HI and PH.

Including HI patients and all reperfusion therapies, we could increase the number of cases respect to previous studies, increasing our statistical power and analyzing the major genetic study performed in this field. In our previous work, we analyzed 1904 patients and in our present study, we were able to analyze 2034 patients.

The differences in these sample sizes are due to the slight increase in the number of cohorts introduced, the generalization of the study to patients who had undergone intra-arterial fibrinolysis or mechanical thrombectomy as a second intention, and the different QC carried out.

Although we did not find statistically significant SNVs after adjusting for multiple comparisons in our discovery cohort, the meta-analysis did allow us to detect rs79770152 with a *p*-value 3.90 × 10^−8^*,* an intronic variant located in the *RP11-362K2.2:RP11-767I20.1* genes, which are uncharacterized genes. We found that the lncRNAs are supposed to likely exert their functions in other genomic locations (trans-regulation) [29].

Another SNV very close to be genome-wide significant was rs13297983 with a *p*-value 6.10 × 10^−8^*,* an intronic variant located in the gene *PCSK5*.

From these leading SNVs of the first ten loci, we can point out that there is one with the most biological evidence to be a regulatory element: rs6686126, an intronic variant located in TGFBR3. In addition, some of these SNVs are eQTL which regulate the expression of different genes in tissues such as the brain, arteries, and peripheral nerves. None of these two SNVs most significant are eQTL or present chromatin interactions regarding the databases available in FUMA.

All the leading SNVs that constituted the top ten most significant variants, followed the same direction of effect in the discovery and replication cohorts. Except rs4348170, which was not present in the discovery cohort. Furthermore, some of the GO terms were related with angiogenesis or neuronal development. This is noteworthy, since the blood vessel is of relevance in the PH and neuronal apoptosis in the prognosis.

Interestingly, several of the genes from the genes included in these loci have been associated in other GWAS studies to aggregated amyloid-β peptide and tau protein such as *PCSK5* or *EIF3H* [30]. *SEMA3A* has been associated with cortical thickness and white matter microstructure measurement [31], parameters related to cognitive impairment. *SEMA3A* gene was also found in the GWAS performed previously by our group (*p*-value: 7.85 × 10^−8^) [10].

We have also found that Alzheimer’s disease, the leading cause of dementia characterized by amyloid-β and tau aggregates, shares a genetic background with a predisposition to PH in patients undergoing reperfusion treatment (raw *p*-value < 0.05). Moreover, we found that WMH also share a genetic background with PH. In previous results from our group, we also observed this genetic correlation with WMH and also with ICH that has not been observed in the current work [10]. We could hypothesize that the lack of this association could be due to the fact that it shares genetic background with HT but not so much with PH, or simply due to a lack of statistical power.

The effect of IVT on overall HT in patients with dementia is controversial in the literature [32]. Some authors conclude that ITV did not increase the risk of HT in the patients with dementia compared to the controls without dementia, that underwent IVT [32].

Our results suggest that dementia might play a role in the development of PH due to Alzheimer’s disease and WMH share a genetic background with PH, although these associations did not remain significant after adjusting for multiple comparisons. Besides, we found SNVs (from the genes *PCSK5*, *EIF3H,* and *SEMA3A)* related to amyloid-β, tau protein, cortical thickness, or WMH. Moreover, the occurrence and localization of cerebral microbleeds (CMBs) associated with IVT-related hemorrhagic complications could indicate an underlying cerebral amyloid angiopathy [33]. This pathology is characterized by the presence of amyloid-β aggregated in the vascular walls of the brain, leading to dementia and a predisposition to ICH. That could indicate that patients who may develop amyloid angiopathy in the future may have an increased risk of HT. However, we did not find a genetic correlation between ICH or ICH subtypes with PH occurrence in our study.

*PCSK5* [34] and *RNA5SP448* [35] has been found to be associated with LDL levels, a molecule that has been shown to promote inflammation [36]. Actually, it has been found that lower LDL cholesterol levels had been associated with HT [3]. *KLF5* has been associated with neutrophil and monocyte count or lymphocyte percentage of leukocytes [37], and *RNA5SP448* with interleukin 12 [38]. Both interleukins and the neutrophil-to-lymphocyte ratio (NLR) have been shown to be a marker associated with inflammation; a high NLR can predict HT [39]. This suggests that inflammation may play an important role in the development of PH. Actually, it has been observed that r-tPA mobilizes immune cells that exacerbate hemorrhagic transformation in stroke [40].

*TGFBR3* has been associated with pulse pressure measurement. Besides, the SNV found with nominal significance: 1:92310874:A:G, an intronic variant located in TGFBR3, has a RegulomeBD score of 2b. In addition, blood pressure variability was found to be correlated with HT [41]. Nevertheless, we failed to find a genetic correlation between SBP and DBP with PH.

It is also worth noting that myosin V binding was the GO term significantly associated with PH. Myosin V is primarily found in the central nervous system serving as neuronal marker [42] and has been linked to recycling endosomes and exocytosis of secretory MMP2 and MMP9 which have been widely associated with TH [43,44,45].

Regarding limitations, one of the most important is the small sample size of both the discovery and replication cohorts, even though it is one of the largest made in this topic. This is probably the root cause of not finding significant SNVs in the discovery cohort. For this reason, to increase our statistical power, we performed the meta-analysis that showed a genome-wide significant SNV and another that was almost significant. Another limitation is the lack of replication in an independent cohort. However, the same direction of effect observed for the most significant SNVs in the discovery and replication cohorts indicates that the results are consistent.

Another limitation is the Spanish origin of all the patients from the discovery cohort, this might make it difficult to generalize the results to other populations. To overcome this limitation, the replication cohort included patients from Poland and Finland. Likewise, the lack of values for the variable of the time elapsed between the onset of symptoms and the administration of treatment may limit our results. Furthermore, the fact that we did not have any patient with mechanical thrombectomy who presented PH limits the generalization of our results to this subgroup of patients. Therefore, studies with a larger sample size, incorporating more variables, and more patients subjected to mechanical thrombectomy will be necessary to establish more robust conclusions.

## 5. Conclusions

With this meta-analysis, we have found a new locus significantly associated with the risk of PH in patients treated with the different types of reperfusion therapies used in the clinical practice. Correlation analysis has shown us shared background genetics between PH and Alzheimer’s disease and WMH. Moreover, the analysis of the most significant genomic loci supports this relationship, as the nearest genes associated with the leading SNVs have been related to aggregated amyloid-β, tau protein, or white matter microstructure. However, also of great interest is that other traits related to these SNVs pointed to the importance that inflammation may play in the risk of developing PH. Further studies are needed to test these hypotheses.

## Figures and Tables

**Table 1:** Discovery cohort.

Study	Total	Cases	Controls	Arrays	Batches	Country
GenoTPA [9]	240	34	180	HumanOmni1-Quad BeadChip (Illumina)	1	Spain
GODS [13]	993	28	342	HumanCoreExome (Illumina)	1	Spain
GRECOS [14]	214	3	0	HumanCoreExome (Illumina)	1	Spain
GENISIS [15]	3220	96	714	HumanCoreExome (Illumina)	5	Spain
Total	4667	161	1236		8	Spain

GenoTPA: Genetic Study in Ischemic Stroke Patients treated with recombinant tissue plasminogen activator (r-tPA); GODS: Genetic contribution to Functional Outcome and Disability after Stroke; GRECOS: Genotyping Recurrence Risk of Stroke study; GENISIS: Genetics of Early Neurological Instability After Ischemic Stroke.

**Table 2:** Replication cohort.

Study	Total	Cases	Controls	Arrays	Batches	Country
GenoTPA [9]	157	36	121	HumanOmni1-Quad BeadChip (Illumina)	1	Spain
BASICMAR	91	8	83	Human Omni Quad 5M (Illumina)	1	Spain
LSGS	45	8	37	Human Omni Quad 5M (Illumina)	1	Belgium
HELSINKI2000	164	12	152	HumanCoreExome (Illumina)	1	Finland
GENISIS [15]	70	2	68	HumanCoreExome (Illumina)	1	Finland
	53	4	49	HumanCoreExome (Illumina)	1	Poland
	484	42	403	HumanCoreExome (Illumina)	1	Spain
Total	1064	112	913		7	Spain

GenoTPA: Genetic Study in Ischemic Stroke Patients treated with recombinant tissue plasminogen activator (r-tPA); BASICMAR: BAse de Datos de ICtus del hospital del MAR; LSGS: Leuven Stroke Genetics Study; HELSINKI2000: Helsinki 2000 Ischemic Stroke Genetics Study; and Genetics of Early Neurological Instability After Ischemic Stroke studies.

**Table 3:** Descriptive analysis of discovery cohort.

Variables (Number of Observations)	Controls (*n* = 1003)	Cases (*n* = 141)	*p*-Value	OR (95% IC)
**Age (*n*= 1142)**	75 ± 16	77 ± 12	6.70 × 10^−2^	
**Sex (*n* = 1144), male**	55% (548/1003)	52% (73/141)	5.29 × 10^−1^	0.89 (0.62–1.29)
**HTN (*n* = 1138)**	64% (634/998)	65% (91/140)	7.79 × 10^−1^	1.07 (0.73–1.58)
**DM (*n* = 1143)**	22% (225/1002)	30% (42/141)	5.64 × 10^−2^	1.46 (0.97–2.19)
**DLP (*n* = 821)**	38% (279/728)	37% (34/93)	8.21 × 10^−1^	0.93 (0.57–1.48)
**AF (*n* = 1140)**	37% (299/999)	38% (53/141)	7.93 × 10^−2^	1.41 (0.96–2.07)
**SH (*n* = 788)**	21% (148/698)	18% (16/90)	3.93 × 10^−1^	0.8 (0.42–1.44)
**NIHSS (*n* = 1141)**	**14 ± 11**	**17 ± 9**	**4.11 × 10^−4^**	
**mRS (*n* = 587)**	**0 ± 1**	**0 ± 1**	**1.84 × 10^−2^**	
**Gly (*n* = 1104)**	119 ± 44	127 ± 49	1.02 × 10^−1^	
**SBP (*n* = 705)**	153 ± 35	158 ± 37	2.50 × 10^−1^	
**DBP (*n* = 731)**	80 ± 20	80 ± 20	4.47 × 10^−1^	
**IA (*n* = 708)**	**5% (28/620)**	**13% (11/88)**	**5.12 × 10^−3^**	**3.01 (1.30–6.55)**
**TM (*n* = 336)**	6% (17/286)	0% (0/50)	8.72 × 10^−2^	0 (0–1.36)
**CES (*n* = 1115)**	46% (451/977)	55% (76/138)	5.57 × 10^−2^	1.43 (0.98–2.08)
**LAS (*n* = 1115)**	**20% (193/977)**	**9% (11/138)**	**3.91 × 10^−4^**	**0.35 (0.17–0.67)**
**SVO (*n* = 1115)**	1% (12/977)	1% (2/138)	6.88 × 10^−1^	1.18 (0.13–5.40)

OR (95% IC): odds ratio (95% confidence interval -CI-). HTN: hypertension, DLP: dyslipidemia, AF: atrial fibrillation, SH: smoking habits, NIHSS: National Institutes of Health Stroke Scale, mRS: modified Rankin Score, Gly: initial glycaemia, SBP: systolic blood pressure, DBP: diastolic blood pressure; IA: intra-arterial fibrinolysis, TM: mechanical thrombectomy, CES: cardioembolic stroke, LAS: large artery atherosclerosis stroke, SVO: small vessel occlusion stroke. For quantitative variables, information is expressed as median ± interquartile range. For categorical variables in frequency (%). Variables significantly associated with PH (*p*-value < 0.05) are highlighted in bold.

**Table 4:** Descriptive analysis of the replication cohort.

Variables (Number of Observations)	Controls (*n* = 819)	Cases (*n* = 76)	*p*-Value	OR (95% IC)
Age (*n* = 895)	72 ± 17	76 ± 11	9.82 × 10^−3^	
Sex (*n* = 895), male	52% (425/819)	53% (40/76)	1	1.03 (0.63–1.7)
DM (*n* = 643)	18% (103/586)	16% (9/57)	8.56 × 10^−1^	0.88 (0.37–1.89)
AF (*n* = 770)	32% (223/700)	49% (34/70)	7.40 × 10^−3^	2.02 (1.19–3.42)
NIHSS (*n* = 895)	11 ± 11	16 ± 8	6.33 × 10^−6^	
Gly (*n* = 464)	120 ± 42	135 ± 52	1.35 × 10^−1^	
CES (*n* = 670)	60% (365/604)	77% (51/66)	7.36 × 10^−3^	2.22 (1.20–4.36)

OR (95% IC): odds ratio (95% confidence interval). AF: atrial fibrillation, NIHSS: National Institutes of Health Stroke Scale, Gly: initial glycaemia, CES: cardioembolic stroke. For quantitative variables, information is expressed as median ± interquartile range. For categorical variables, in frequency (%).

**Table 5:** The ten genomic loci with its leading SNV.

ID	Rs	*p*-Value	Beta (SE)	MAF	Nearest Gene	Func	RDB	eQTL	GO Terms
12:59127963:A:G	rs79770152	Metal: 3.90 × 10^−8^ DC: 2.18 × 10^−5^ RC: 3.79 × 10^−5^	0.0895 (0.0152) (++)	9%	*RP11-362K2.2:RP11-767I20.1*	ncRNA (intronic)	7		
9:78563802:G:T	rs13297983	Metal: 6.10 × 10^−8^ DC: 7.80 × 10^−7^ RC: 1.00 × 10^−3^	0.0968 (0.0166) (++)	7%	*PCSK5*	Intronic	7		Renin secretion into blood stream, proteolysis, heart development
13:73655521:G:T	rs1537385	Metal: 1.69 × 10^−7^ DC: 1.11 × 10^−4^ RC: 4.09 × 10^−5^	−0.1096 (0.0195) (--)	4%	*KLF5*	Intergenic	3a		Angiogenesis
10:10130938:A:G	rs35246078	Metal: 1.87 × 10^−7^ DC: 1.50 × 10^−4^ RC: 3.57 × 10^−5^	0.1091 (0.0195) (++)	7%	*RP5-933E2.1*	Intergenic	6		
4:148508838:G:T	rs61170156	Metal: 1.28 × 10^−6^ DC: 2.20 × 10^−5^ RC: 1.45 × 10^−3^	0.0670 (0.0129) (++)	17%	*RP11-752L20.5*	ncRNA (intronic)	5	TMEM184C (thyroid), GPRC5C, CD300C, BTBD17, KIF19, FDXR, MRPS7	
1:92310874:A:G	rs6686126	Metal: 1.37 × 10^−6^ DC: 9.87 × 10^−3^ RC: 3.13 × 10^−6^	0.0868 (0.0167) (++)	7%	*TGFBR3*	Intronic	2b		Blood vessel development, heart morphogenesis, organ regeneration
15:45737253:A:C	rs72711259	Metal: 1.58 × 10^−6^ DC: 2.12 × 10^−2^ RC: 6.62 × 10^−7^	0.0731 (0.0142) (++)	12%	*C15orf48*	Intronic	7	SLC28A2 (rectum, colon, esofagus and gastroesophagus junction, tibial nerve, testis, thyroid, cervical spinal cord), SQRDL (thyroid), SLC30A4, SHF (cervical spinal cord), DUOX1 (cerebellum), SORD, SPATA5L1 (adipose tissue, whole blood, artery tibial, esofagus and gastroesophagus junction, skeletal muscle, thyroid), SLC28A2, SPATA5L1, RP11-96O20.4, GATM (artery tibial, skeletal muscle), TRIM69 (thyroid)	Nucleus, mitochondrion
17:72393744:A:G	rs4348170	Metal: 1.60 × 10^−6^ DC: - RC: 2.55 × 10^−7^	0.1077 (0.0209) (?+)	10%	*RNA5SP448*	Intergenic	NA	BTBD17, FDXR (brain cortex), CD300C, FDXR, GPRC5C (adrenal gland), KIF19, MRPS7 (liver)	
7:83857204:C:T	rs7802925	Metal: 2.09 × 10^−6^ DC: 4.73 × 10^−6^ RC: 6.89 × 10^−3^	−0.0907 (0.0178) (--)	6%	*SEMA3A*	Intronic	5		Apoptotic process, neuron migration, nerve development
8:117535199:C:T	rs16888486	Metal: 2.19 × 10^−6^ DC: 2.71 × 10^−3^ RC: 3.10 × 10^−5^	−0.1023 (0.0201) (++)	6%	*EIF3H*	Intergenic	7	UTP23 (whole blood)	Gene expression, extracellular vesicular exosome

ID: SNV identifier; rs: RefSNP; Beta (SE): β coefficient and standard error, between brackets the direction of the SNV in the discovery and replication cohort; MAF: minor allele frequency; Func: functional consequence of the SNV on the gene obtained from ANNOVAR; RDB: RegulomeDB score which is the categorical score (from 1a to 7), 1a is the highest score that the SNV has the most biological evidence to be regulatory element; eQTL: expression quantitative trait loci, here appears the gene which expression the SNV modifies; GO terms: the most relevant gene ontology terms. +: positive effect of the β coefficient; -: negative effect of the β coefficient; ?: the SNV was not evaluated; the first symbol corresponds to discovery and the second to replication cohorts.

**Table 6:** Results of the genetic correlation (GNOVA).

Trait	Rho	Rho SE	Corr	*p*-Value
**Alzheimer’s Disease**	**0.049**	**0.021**	**0.200**	**2.15 × 10^−2^**
**White Matter Hyperintensities**	**−0.100**	**0.047**	**−2.257**	**3.46 × 10^−2^**
**Deep ICH**	0.089	0.80	0.141	2.66 × 10^−1^
**ICH**	0.070	0.082	0.199	3.03 × 10^−1^
**SVO**	−0.006	0.008	−0.067	4.90 × 10^−1^
**SBP**	−0.007	0.013	−0.021	6.06 × 10^−1^
**Lobar ICH**	0.031	0.080	0.189	6.96 × 10^−1^
**CES**	−0.002	0.008	−0.021	7.96 × 10^−1^
**AIS**	−0.002	0.008	−0.017	8.32 × 10^−1^
**DBP**	−0.003	0.013	−0.009	8.41 × 10^−1^
**LAS**	−0.001	0.009	−0.006	9.46 × 10^−1^
**AS**	−0.0005	0.008	−0.005	2.66 × 10^−1^

Rho: the genetic covariance estimate; rho SE: standard error of the estimate of rho; Corr: the genetic correlation estimate. ICH: intracerebral hemorrhage; SVO: small vessel occlusion stroke; SBP: systolic blood pressure; CES: cardioembolic stroke; AIS: any ischemic stroke; DBP: diastolic blood pressure; LAS: large artery atherosclerosis stroke; AS: all strokes. Traits with *p*-values < 0.05 are highlighted in bold.

## Data Availability

The data presented in this study are available on request from the corresponding author.

## Supplementary Materials

The following are available online at https://www.mdpi.com/article/10.3390/jcm10143137/s1, Figure S1: Manhattan and QQ plot of the discovery cohort; Figure S2: Manhattan and QQ plot of the discovery cohort; Table S1: SNVs belonging to the genomic locus with the leading SNP being significant at GWAS level; Table S2: Description of the GWAS significant locus and the 28 nominal significant loci; and Table S3: Top ten of the most significant curated gene sets and gene ontology terms obtained from MsigDB.
