# Supplementary material for: RP11-362K2.2:RP11-767I20.1 Genetic Variation Is Associated with Post-Reperfusion Therapy Parenchymal Hematoma. A GWAS Meta-Analysis
Source: J Clin Med. 2021 Jul 16;10(14):3137. doi: 10.3390/jcm10143137 (PMC8305811; doi:10.3390/jcm10143137)
Supplement: Supplementary file 1 [file jcm-10-03137-s001.zip › jcm-1271745-supplementary.pdf]

Supplementary Material

SUPPLEMENTAL METHODS

1. Description of the studies included in the discovery and replication cohorts

1.1. The Genetic study in ischemic stroke patients treated with tPA (GenoTPA)

Consecutive Caucasian patients with acute ischemic stroke who were admitted to the emergency room and received recombinant tissue-type plasminogen activator (r-tPA) within 4.5 hours of symptom onset were recruited. Patients were enrrolled at Spanish hospitals (Vall d'Hebron University Hospital, Hospital Clinic, Hospital Universitari de Girona Doctor Josep Trueta, Hospital de la Santa Creu i Sant Pau, Hospital Universitari Germans Trias I Pujol, Hospital Universitari del Mar, Hospital de Basurto) between 2002 to 2012. The study protocol was approved by the Ethics Committee of each center, all patients or relatives signed the informed consent.

Patients were identified by medical evaluation at emergency room arrival; stroke diagnosis was performed by trained neurologists and confirmed by neuroimaging. There were no exclusion criteria regarding age, sex or ethnicity. Follow-up CT scan at 24 hours after onset of symptoms or if neurological worsening occurred were performed and was classified according to European Cooperative Acute Stroke Study (ECASS) [1].

1.2. Genetic contribution to functional Outcome and Disability after Stroke (GODS)

Subjects with diagnosis of acute ischemic stroke, baseline NIHSS assessment >4, and functional outcome 3 at 90 days were selected from the Spanish Stroke Genetics Consortium (GeneStroke). Diagnosis of ischemic stroke was based on neurological symptoms and neuroimaging. Subjects were excluded whether aged <18 years old, concomitant pathology or recurrent stroke during the 3 months follow-up occurs, and posterior vascular territory infarction or lacunar stroke were detected. Each one of the Ethics Committees of the participating centers approved the study, and all patients or relatives signed the informed consent [2].

Acute ischemic stroke participants were recruited at Spanish centers: Hospital Universitari del Mar (Barcelona), Hospital Universitari Son Espases (Mallorca), Vall d' Hebron University Hospital (Barcelona), Hospital Clínic (Barcelona), Hospital Universitari de Girona Doctor Josep Trueta (Girona), Hospital de la Santa Creu i Sant Pau (Barcelona), Hospital Universitari Germans Trias I Pujol (Can Ruti) and Hospital de Basurto (Bilbao). All the selected patients received intravenous thrombolytic therapy.

1.3. Genotyping Recurrence Risk of Stroke (GRECOS)

Consecutive Caucasian patients, aged >18 years old, with a first ischemic stroke that were admitted to the emergency department of 23 Spanish Hospitals were recruited. Stroke diagnosis was performed by trained neurologists and confirmed by neuroimaging. For the present study, we selected patients treated with r-tPA, who were enrolled at the Vall d' Hebron University Hospital.

Patients with modified Rankin Scale (mRS) at discharge ≥4, with a life expectancy lower than one year at the time of inclusion, and patients participating in a clinical trial of secondary prevention of stroke were excluded. On basis of their experience and information provided by other physicians, the neurologists decided whether to include or exclude each patient from the study. Risk factors, clinical evolution and radiological data were collected from the clinical records during hospitalization. One-year follow-up were performed to record the occurrence of ischemic stroke recurrence or global vascular recurrences. Moreover, each participant received a phone call and standard questionnaires to capture follow-up clinical and demographic every 3 months. The study was approved by the ethics committee, and all patients or their relatives gave informed written consent.

1.4. Genetics of Early Neurological Instability after Ischemic Stroke (GENISIS)

GENISIS is an international project which aim is to capture the variety of mechanisms related to brain injury and early recovery. The study consisted in ischemic stroke patients, with neurological evaluation (NIHSS) within 6 hours from onset of symptoms and 24-hours follow-up. The stroke diagnosis was performed by trained neurologists and confirmed by neuroimaging. Participants had to be over the age of 18 years, have provided informed consent, and do not underwent endovascular thrombectomy. Local Institutional Review Boards approved the enrolling and the data collection in each site [3].
Patients treated with r-tPA and information of hemorrhagic transformation were selected from Spain recruitment sites: Vall d'Hebron University Hospital, Hospital Universitari Germans Trias I Pujol, Hospital Universitari del Mar, Hospital de la Santa Creu i Sant Pau, Mutua de Terrassa University Hospital, Hospital Universitario de Albacete, Hospital Clínico Universitario Valladolid, Hospital Clínico Universitario de Santiago, Hospital Virgen del Rocío y Virgen de la Macarena, Hospital Universitari Son Espases, Hospital Clinic.

1.5. BASe de datos de ICtus del hospital del Mar (BASICMAR)

The Stroke database from Hospital del Mar (BASICMAR) is an ongoing prospective study of all acute strokes assessed since 2005 at the IMIM-Hospital Universitari del Mar (Barcelona, Spain). The hospital serves three of the ten districts in Barcelona, Spain providing medical care for a population of 339,196. Patients with first-ever and recurrent stroke are included. There are no exclusion criteria regarding age, sex or ethnicity. All participants provided written consent. Clinical evaluation and neuroimaging were assessed by trained neurologist. Patients with acute infarct confirmed radiographically and treated with thrombolysis therapy were selected.

1.6. Leuven Stroke Genetics Study (LSGS)

Patients admitted to the Stroke Unit of the University Hospitals at Leuven, Belgium between 2005 and 2009 were enrolled. Subjects were European descent, aged 18 years or older with acute cerebral ischemia, defined as a clinical stroke with imaging confirmation. All participants underwent brain imaging and a standardized protocol including lab examination, carotid ultrasound, and cardiac examination8 . Those who received r-tPA and with 24 hours follow-up CT scan were select. Hemorrhagic transformation subtype was determining following the European Cooperative Acute Stroke Study (ECASS) [1]. Written informed consent was obtained from all subjects.

1.7. Helsinki 2000 Ischemic Stroke Genetics Study (HELSINKI2000)

The study was designed for investigating genetic factors underlying ischemic stroke in a Finnish population and in the long-term to be incorporated to multicenter multinational similar datasets. This study received approval from local ethics committee (October 27, 2010; and Amended June 27, 2016). All ischemic stroke cases in this study were recruited from the Helsinki University Central Hospital, which is the only neurological emergency unit for a population of 1.5 million inhabitants [4]. Only patients with positive neuroimaging findings for a new-onset brain infarction were recruited following written informed consent. All included subjects are of Finnish Caucasian origin.

2. Description of the variables

Hypertension: defined as two measures on different days with blood pressure exceeding 140/90 mmHg or taking antihypertensive treatment.
Diabetes mellitus (DM): defined as basal glycemia in venous plasma ≥126 mg/dl, 2-h post-load plasma glycemia ≥200 mg/dl or HbA1c ≥ 6.5% or taking antidiabetic treatment.
Dyslipidemia: elevated low-density lipoprotein (LDL) cholesterol, low high-density lipoprotein (HDL) cholesterol, excess lipoprotein "a", hypertriglyceridemia, or mixed lipid disorders.
Atrial fibrillation (AF): the subject carries a pre-existing diagnosis of AF, and may or may not be anticoagulated; or the subject has an EKG in the emergency room showing AF.

Smoking habits
National Institutes of Health Stroke Scale (NIHSS) (0-42 points) assessed at the initial clinical evaluation.
Modified Rankin Score (mRS): this is a scale designed to evaluate disability after a stroke, ranging from 0 to 6: 0: no disability; 1: disability that does not limit daily life; 2: disability that limits daily life, but the patient is still independent; 3: disability that makes the patient dependent; 4: patient dependent and unable to walk without assistance; 5: patient bedridden; 6: death.
Glucose, a measurement of the blood glucose level in mg/dl obtained in the acute setting during the initial clinical evaluation or before rtPA bolus.
Systolic and diastolic blood pressure (SBP/DBP), was measured in mmHg at the time of admission to the emergency department, prior to r-tPA bolus.
Stroke etiologic subtypes were classified according to the Trial of ORG 10172 in Acute Stroke Treatment (TOAST) [5]:
Cardioembolism (CES): patients with arterial occlusions presumably due to an embolus arising in the heart.
Large Artery Atherosclerosis (LAS): ischemic stroke due to stenosis >50% or occlusion of a major brain artery or branch cortical artery, presumably due to atherosclerosis.
Small Artery Occlusion (SVO): patients with normal CT/MRI examination or a relevant brain stem or subcortical hemispheric lesion with a diameter of less than 1.5 cm that should have the traditional lacunar syndrome.
Other Determinied Etiology (ODT): patients with rare causes of stroke, such as nonatherosclerotic vasculopathies, hypercoagulable states, or hematologic disorders.
Undetermined Etiology (UND) includes: patients with two or more potential causes of stroke so that the physician is unable to make a final diagnosis, a negative evaluation (unknown etiology), or incomplete evaluation.

3. Quality Controls

3.1. Discovery cohort

The quality controls (QC) were applied using PLINK v1.9 and v2.0 and KING software. The principal pre-imputational QC analysis carried out (per array) were:

- Removing genetic variants that are missing in a determined proportion of individuals calculating the genotyping call rate and through visual inspection of the histogram of frequencies. For GenoTPA those with 3% of missings, GODS and GRECOS 5% and GENISIS 1%.
- Removing subjects who have a determined rate of genotype missingnesss, taking into account the visual inspection of the histogram of frequencies. For GenoTPA and GRECOS those individuals with 3% of missings, GODS 5% and GENISIS 2%. Total of individuals removed: 122.
- Remove non biallelic SNV, ambiguous SNV to avoid unreliable strand orientation, monomorfic or duplicated SNV.
- Remove individuals with difference between the assigned sex and the sex determined based on the genotype (sex discordance) or without sex information. Total of individuals removed: 279.
- Remove family members or duplicate samples. Calculating identity by descent (IBD) of all sample pairs, using independent SNV and selecting those pairs of individuals with a pi-hat >0.2. Total of individuals removed: 35.
- Remove non-European individuals. For the identification of population substructure, a multidimensional scaling (MDS) was performed with the KING software (https://people.virginia.edu/~wc9c/KING/kingpopulation.html). Total of individuals removed: 99.

- Remove SNV that violates Hardy–Weinberg (dis)equilibrium (HWE) law, and therefore genotype frequencies were significantly different than expected. We established two thresholds, for SNV in controls, those with a p-value <1x10-6 and for cases, <1x10-10 [6].

After these initial QCs, the merge of all the arrays that would constitute the Discovery cohort was carried out in order to perform:

- Remove family members or duplicate samples. Calculating IBD of all sample pairs, using independent SNV and selecting those pairs of individuals with a pi-hat >0.2.
- Check that the origin of all individuals was still estimated to be European.
- Remove SNV with minor allele count (MAC) less than one.
- Exclude individuals with high or low heterozygosity rates. We considered outliers to be approximately 1% of individuals at the extremes of the F coefficient distribution (deviations from the median greater than six times the interquartile range).
- Remove SNV that violates Hardy–Weinberg (dis)equilibrium (HWE) law, and therefore genotype frequencies are significantly different than expected. We stablised two thresholds, for SNV in controls, those with a p-value <1x10-6 and for cases, <1x10-10 [6].
- A total of 279 individuals were removed in this second step.

Subsequently, individuals and SNVs were removed from each array, which did not pass QC in this second step. After that, studies genotyped on the same platforms were combined for the imputation analysis.

2.2. Replication cohort

The participants included in the replication cohort were part of the Genetic Study in Ischemic Stroke Patients treated with tPA (GenoTPA) [7], BAse de Datos de ICtus del hospital del MAR (BASICMAR) (Stroke database of the Hospital del Mar) [8], Leuven Stroke Genetics Study (LSGS) [9], Helsinki 2000 Ischemic Stroke Genetics Study and Genetics of Early Neurological Instability After Ischemic Stroke (GENISIS) [10] studies.

This genetic information was divided into two parts. Replication I with GenoTPA, BASICMAR, LSGS and HELSINKI2000 patients, and who constituted the replication cohort in the study conducted by Carreta et al. [11]; and replication II with one batch of GENISIS patients. Both parts were pooled to obtain a single replication and QC analysis. Both parts were already imputed and QC were also performed.

Once imputed, the two parts were merged to eliminate any family members and duplicates that might exist, calculating IBD of all sample pairs and selecting those pairs of individuals with a pi-hat >0.2.

After this process, the discovery and replication cohorts were merged to remove duplicate samples from the replication cohort, calculating IBD of all sample pairs and selecting those pairs of individuals with a pi-hat >0.8.

2.3. Genome-wide association analysis and meta-analysis

We performed a linear regression-based association analysis using fastGWAS [12]. Those SNV with minor allele count (MAC) <6 were subsequently removed. For discovery cohort we adjusted for the first two principal components (PC), age and the variables remaining significant in the multivariable logistic regression (p-value <0.05) and that we had information on the replication cohort: NIHSS. For replication cohort the analysis was adjusted for the three first PC, and the same clinical variables than in the discovery analysis: age and NIHSS. The different number of PC depended on visual inspection; they were added until they no longer differentiated groups within the cohorts (Figure 1).

SUPPLEMENTARY FIGURES

Figure S1. Manhattan and QQ plot of the discovery cohort. In the Manhattan plot, SNVs were represented by dots and plotted based on their genome-wide association study p-values. Redline shows genome-wide significance (p-value $<5\times10^{-8}$). In the QQ plot of the p-values obtained after the association testing, the x-axis represents the expected $-\log_{10}$ - p-value under the null hypothesis and lambda is the median of the resulting chi-squared test statistics divided by the expected median of the chi-squared distribution under the null hypothesis.

Figure S2. Manhattan and QQ plot of the discovery cohort. In the Manhattan plot, SNVs were represented by dots and plotted based on their genome-wide association study p-values. Redline shows genome-wide significance (p-value $<5\times10^{-8}$). In the QQ plot of the p-values obtained after the association testing, the x-axis represents the expected $-\log_{10}$ - p-value under the null hypothesis and lambda is the median of the resulting chi-squared test statistics divided by the expected median of the chi-squared distribution under the null hypothesis.

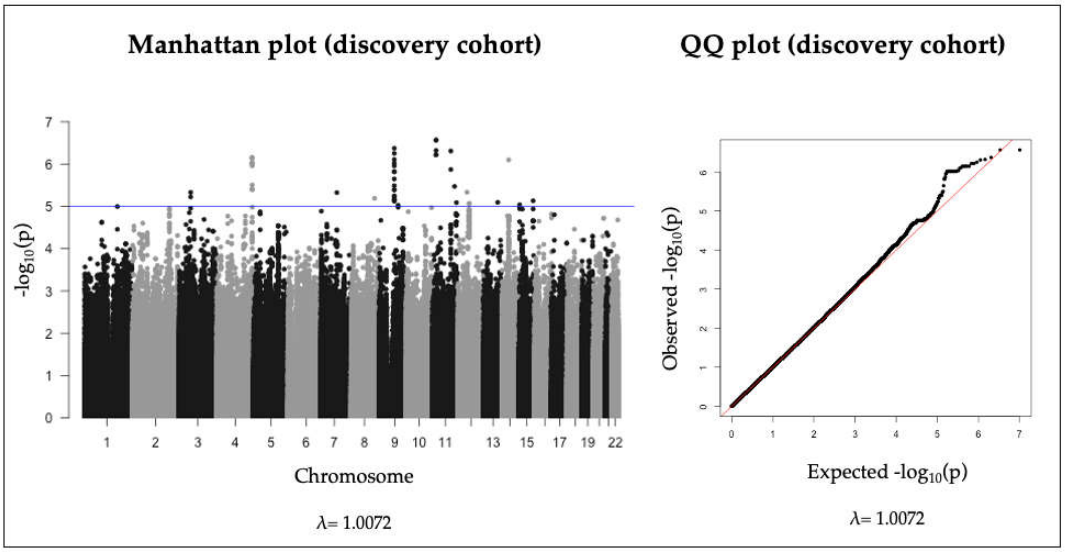

Figure S1. Manhattan and QQ plot of the discovery cohort. In the Manhattan plot, SNVs were represented by dots and plotted based on their genome-wide association study p-values. Redline shows genome-wide significance (p-value $<5\times10^{-8}$). In the QQ plot of the p-values obtained after the association testing, the x-axis represents the expected $-\log_{10}$ - p-value under the null hypothesis and lambda is the median of the resulting chi-squared test statistics divided by the expected median of the chi-squared distribution under the null hypothesis.

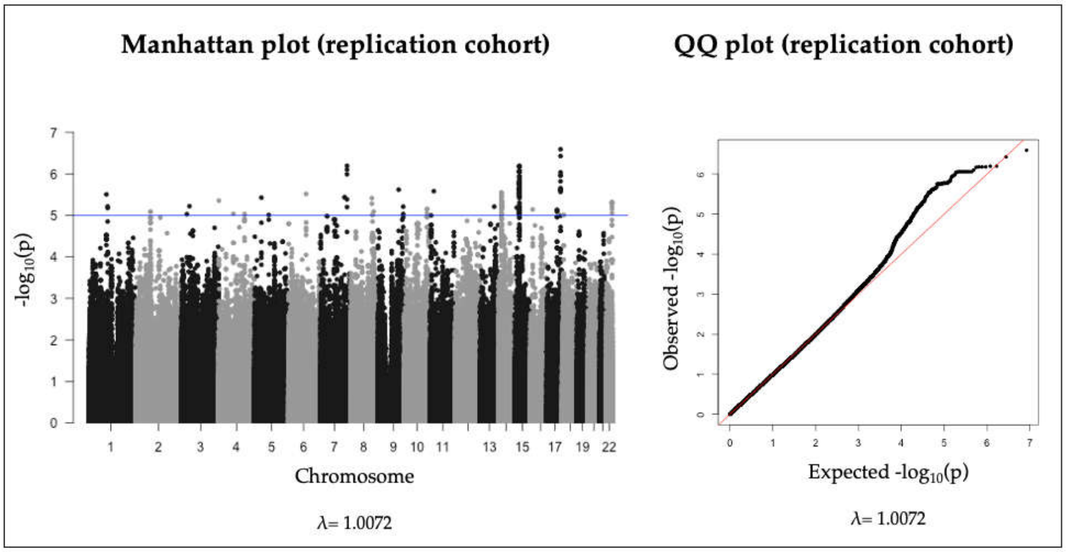

Figure S2. Manhattan and QQ plot of the discovery cohort. In the Manhattan plot, SNVs were represented by dots and plotted based on their genome-wide association study p-values. Redline shows genome-wide significance (p-value $<5\times10^{-8}$). In the QQ plot of the p-values obtained after the association testing, the x-axis represents the expected $-\log_{10}$ - p-value under the null hypothesis and lambda is the median of the resulting chi-squared test statistics divided by the expected median of the chi-squared distribution under the null hypothesis.

SUPPLEMENTARY TABLES

Table S1. SNVs belonging to the genomic locus with the leading SNV being significicant at GWAS level. These SNVs are inside the gene RP11-362K2.2:RP11-767I20.1, in chromosome 12, being all of them intronic variants.

ID	MAF	p-value	Beta (SE)	RDB	r^2
12:59127963:A:G	9%	$3.90x10^{-8}$	0.0895 (0.0152)	7	1
12:59133424:A:C	9%	$3.90x10^{-8}$	0.0895 (0.0152)	6	0.996
12:59127305:C:T	9%	$3.90x10^{-8}$	-0.0895 (0.0152)	7	0.999
12:59127259:C:T	9%	$3.90x10^{-8}$	-0.0895 (0.0152)	7	0.999
12:59121881:A:G	9%	$3.90x10^{-8}$	-0.0895 (0.0152)	6	0.996
12:59120715:C:T	9%	$3.90x10^{-8}$	0.0895 (0.0152)	5	0.996
12:59130097:C:T	9%	$3.94x10^{-8}$	0.0895 (0.0152)	6	0.996
12:59134318:A:C	11%	$8.02x10^{-8}$	-0.0834 (0.0145)	6	0.817
12:59133663:C:T	11%	$8.02x10^{-8}$	0.0834 (0.0145)	7	0.820
12:59129771:A:G	11%	$8.02x10^{-8}$	0.0834 (0.0145)	6	0.820
12:59122003:A:C	11%	$8.02x10^{-8}$	0.0834 (0.0145)	-	0.820
12:59121258:A:G	11%	$8.02x10^{-8}$	0.0834 (0.0145)	5	0.820
12:59114375:A:G	11%	$8.80x10^{-8}$	-0.0826 (0.0144)	5	0.815
12:59111515:C:T	11%	$8.89x10^{-8}$	-0.0830 (0.0145)	7	0.815
12:59109954:C:T	11%	$9.40x10^{-8}$	-0.0828 (0.0144)	7	0.815
12:59128060:A:G	11%	$9.45x10^{-8}$	-0.0828 (0.0144)	7	0.821
12:59133027:A:C	11%	$1.02x10^{-7}$	0.0826 (0.0144)	6	0.818
12:59128693:A:G	11%	$1.02x10^{-7}$	-0.0826 (0.0144)	7	0.818
12:59124921:G:T	11%	$1.02x10^{-7}$	-0.0826 (0.0144)	6	0.821
12:59122775:A:G	11%	$1.02x10^{-7}$	0.0826 (0.0144)	7	0.818
12:59111385:A:G	8%	$1.12x10^{-7}$	-0.0882 (0.0155)	7	0.904
12:59135562:C:T	9%	$1.54x10^{-7}$	-0.0857 (0.0152)	7	0.998
12:59135055:C:T	11%	$1.54x10^{-7}$	0.0857 (0.0152)	5	0.993
12:59117994:C:T	11%	$1.76x10^{-7}$	0.0807 (0.0144)	-	0.818
12:59119206:C:T	11%	$1.77x10^{-7}$	0.0812 (0.0145)	-	0.819
12:59135902:C:T	11%	$2.28x10^{-7}$	-0.0801 (0.0144)	-	0.818
12:59136639:A:G	11%	$4.97x10^{-7}$	0.0783 (0.0145)	6	0.818
12:59096079:A:T	8%	$5.60x10^{-7}$	-0.0817 (0.0152)	6	0.868
12:59087463:C:T	8%	$5.60x10^{-7}$	0.0817 (0.0152)	7	0.867
12:59096530:A:C	8%	$6.91x10^{-7}$	0.0806 (0.0151)	6	0.868
12:59084825:A:G	8%	$6.94x10^{-7}$	0.0810 (0.0152)	6	0.863
12:59082179:A:G	8%	$6.94x10^{-7}$	0.0810 (0.0152)	7	0.847

12:59095169:A:G	8%	7.52x10^{-7}	0.0806 (0.0152)	7	0.868
12:59094337:A:G	8%	7.52x10^{-7}	0.0806 (0.0152)	6	0.868
12:59085454:C:T	8%	7.52x10^{-7}	0.0806 (0.0152)	7	0.864
12:59097221:C:T	8%	8.94x10^{-7}	-0.0795 (0.0151)	6	0.865
12:59079775:A:G	8%	9.02x10^{-7}	0.0800 (0.0152)	6	0.826
12:59080855:A:G	8%	9.07x10^{-7}	0.0800 (0.0152)	7	0.844
12:59082262:C:T	8%	9.29x10^{-7}	0.0794 (0.0151)	7	0.854
12:59089022:C:T	8%	1.16x10^{-6}	-0.0790 (0.0151)	7	0.855
12:59088248:C:T	8%	1.19x10^{-6}	-0.0789 (0.0151)	6	0.852
12:59096175:A:C	10%	1.29x10^{-6}	.0.0734 (0.0141)	7	0.781
12:59098224:A:G	8%	1.032x10^{-6}	0.0791 (0.0152)	5	0.870
12:59088582:A:G	11%	1.45x10^{-6}	-0.0730 (0.0141)	7	0.770
12:59102830:A:G	10%	1.53x10^{-6}	-0.0731 (0.0142)	7	0.780
12:59101061:A:G	10%	1.53x10^{-6}	-0.0731 (0.0142)	7	0.780
12:59108988:A:T	8%	1.76x10^{-6}	-0.0782 (0.0152)	6	0.870
12:59107756:C:G	8%	1.78x10^{-6}	0.0781 (0.0152)	6	0.870
12:59104744:C:T	8%	1.78x10^{-6}	-0.0781 (0.0152)	3a	0.870
12:59102054:A:C	8%	1.78x10^{-6}	-0.0781 (0.0152)	7	0.870
12:59101351:C:T	8%	1.78x10^{-6}	0.0781 (0.0152)	7	0.870
12:59101279:C:T	8%	1.78x10^{-6}	0.0781 (0.0152)	7	0.870
12:59091530:C:G	8%	2.55x10^{-6}	0.0758 (0.0150)	6	0.850
12:59109359:A:T	8%	3.51x10^{-6}	0.0759 (0.0152)	6	0.871
12:59104495:A:T	8%	1.17x10^{-4}	0.0928 (0.0224)	6	0.867
12:59138934:A:T	9%	1.63x10^{-4}	0.0869 (0.0215)	7	0.675
12:59099848:G:T	10%	2.57x10^{-3}	-0.0629 (0.0194)	7	0.781

* ID: SNV identifier; Beta (SE): beta coefficient and standard error; MAF: minor allele frequency; RDB: RegulomeDB score which is the categorical score (from 1a to 7), 1a is the highest score that the SNV has the most biological evidence to be regulatory element; r^2: the maximum r^2 of the SNV with the independent significant SNV of the genomic locus.

Table S2. Description of the GWAS significant locus and the 28 nominal significant loci.

Genomic Locus	Lead SNV	chr	p	start	end	n SNVs	n GWAS SNVs	n Ind Sig SNVs	n Lead SNVs
1	1:92310874:A:G	1	$1,37x10^{-4}$	92245298	92321650	8	5	1	1
2	2:1047076:C:G	2	$7,46x10^{-5}$	934215	1047076	83	70	1	1
3	3:60711009:A:G	3	$6,36x10^{-5}$	60711009	60711009	1	1	1	1
4	4:137763599:A:G	4	$6,31x10^{-5}$	137733650	137776232	9	9	1	1
5	4:148508838:G:T	4	$1,28x10^{-5}$	148504101	148518896	9	9	1	1
6	4:151006540:C:T	4	$7,63x10^{-5}$	151006540	151123991	6	6	1	1
7	4:183928464:A:T	4	$5,32x10^{-5}$	183896784	183929581	42	39	1	1
8	7:3043406:G:T	7	$9,40x10^{-5}$	3039547	3060600	9	9	1	1
9	7:34531493:C:G	7	$7,72x10^{-5}$	34530444	34539492	12	11	1	1
10	7:83857204:C:T	7	$2,09x10^{-5}$	83803592	83953457	45	41	1	1
11	7:140831341:A:C	7	$3,57x10^{-5}$	140831341	140905396	6	6	1	1
12	8:83734479:A:G	8	$4,85x10^{-5}$	83729215	83734479	4	4	1	1
13	8:117535199:C:T	8	$2,19x10^{-4}$	117476379	117577255	30	27	1	1
14	8:120085494:C:T	8	$4,94x10^{-5}$	119868659	120199903	61	55	2	1
15	9:78563802:G:T	9	$6,10x10^{-3}$	78548817	78585925	19	18	1	1
16	10:10130938:A:G	10	$1,87x10^{-3}$	10084061	10157987	45	42	1	1
17	12:59127963:A:G	12	$3,90x10^{-3}$	59079775	59138934	65	57	1	1
18	13:73655521:G:T	13	$1,69x10^{-4}$	73655521	73655521	1	1	1	1
19	13:109515199:A:G	13	$2,76x10^{-5}$	109506410	109538679	13	11	1	1
20	14:29111778:A:G	14	$8,72x10^{-5}$	29080887	29160076	18	16	1	1
21	14:55770381:A:T	14	$4,69x10^{-5}$	55546461	55882548	250	199	1	1
22	14:59504942:A:G	14	$8,14x10^{-3}$	59469289	59505868	10	10	1	1
23	15:45737253:A:C	15	$1,58x10^{-5}$	45571923	45947393	180	153	1	1
24	17:6286918:C:T	17	$8,35x10^{-5}$	6286918	6287147	2	2	1	1

25	17:72393744:A:G	17	$1,60 \times 10^{-5}$	72383329	72425183	35	21	1	1
26	22:20168295:G:T	22	$8,32 \times 10^{-5}$	20156744	20169499	25	23	1	1
27	22:33041385:C:G	22	$3,05 \times 10^{-5}$	33039316	33041767	9	6	1	1
28	22:43127465:A:G	22	$4,94 \times 10^{-5}$	43111670	43145466	21	20	1	1
29	22:49050773:C:T	22	$2,74 \times 10^{-5}$	49040844	49059969	42	38	1	1

Genomic Locus: Index of genomic rick loci; Lead SNVs: lead SNVs in the genomic locus consists of chr:position:allele1:allele2 where alleles are alphabetically ordered; chr: chromosome of top lead SNV; p: p-value of top lead SNV; start: Start position of the locus; end : End position of the locus; n SNVs: Number of unique candidate SNVs in the genomic locus, including non-GWAS-tagged SNVs; n GWAS SNVs: Number of the GWAS-tagged candidate SNVs within the genomic locus. This is a subset of "n SNVs"; n Ind Sig SNVs: Number of the independent significant SNVs in the genomic locus; n Lead SNPs : The number of lead SNPs in the genomic locus.

Table S3. Top ten of the most significant curated gene sets and gene ontology terms obtained from MsigDB.

	n genes	Beta	SE	p-value	Adj. p-value
Myosin V binding	16	0.94	0.03	$1.32x10^{-7}$	$2.04x10^{-3}$
Mitotic DNA replication checkpoint	10	1.16	0.03	$1.77x10^{-5}$	$2.74x10^{-1}$
Long cahin fatty acid import across plasma membrane	5	1.31	0.02	$1.91x10^{-5}$	$2.96x10^{-1}$
Schaeffer prostate development 48hr dn	381	0.17	0.02	$8.98x10^{-4}$	1
Bernard PPAPDC1B targets dn	54	0.41	0.02	$1.09x10^{-4}$	1
Nuclear cyclin dependent protein kinase holoenzyme complex	12	0.68	0.02	$1.34x10^{-4}$	1
Cytoskeletal protein binding	890	0.10	0.02	$1.83x10^{-4}$	1
Aspartate family amino acid catabolic process	22	0.57	0.02	$2.39x10^{-4}$	1
Bredemeyer rag signaling via atm not via nfkb dn	37	0.49	0.02	$4.94x10^{-4}$	1
Anterograde dendritic transport	5	1.26	0.02	$4.98x10^{-4}$	1

* n genes: number of genes; SE: standard error; adj. p-value: p-value adjusted by Bonferroni method.

SUPPLEMENTARY REFERENCES

1. V Larrue, R von Kummer, G del Zoppo, E.B. Hemorrhagic Transformation in Acute Ischemic Stroke. Potential Contributing Factors in the European Cooperative Acute Stroke Study. *Stroke* **1997**, *28*, 957–960, doi:10.1161/01.str.28.5.957.
2. Mola-Caminal, M.; Carrera, C.; Soriano-Tárraga, C.; Giralt-Steinhauer, E.; Díaz-Navarro, R.M.; Tur, S.; Jiménez, C.; Medina-Dols, A.; Cullell, N.; Torres-Aguila, N.P.; et al. PATJ Low Frequency Variants Are Associated with Worse Ischemic Stroke Functional Outcome: A Genome-Wide Meta-Analysis. *Circ. Res.* **2019**, *124*, doi:10.1161/CIRCRESAHA.118.313533.
3. Laura Ibanez, PhDa, Laura Heitsch, MDb, c, Umber Dube, BSca, Fabiana H. G. Farias, PhDa, John Budde, ABa, Kristy Bergmann, BSca, Rich Davenport, BSca, Joseph Bradley, BSca, Caty Carrera, MScd, Janne Kinnunen, MDe, Hanne Sallinen, MDe, Daniel Strbian, MD, P.I.S.G.C. and the M.C. Overlap in the Genetic Architecture of Stroke Risk, Early Neurological Changes and Cardiovascular Risk Factors. **2019**, *50*, 1339–1345, doi:10.1161/STROKEAHA.118.023097.
4. Putaala, J.; Metso, A.J.; Metso, T.M.; Konkola, N.; Kraemer, Y.; Haapaniemi, E.; Kaste, M.; Tatlisumak, T. Analysis of 1008 Consecutive Patients Aged 15 to 49 with First-Ever Ischemic Stroke the Helsinki Young Stroke Registry. *Stroke* **2009**, *40*, 1195–1203, doi:10.1161/STROKEAHA.108.529883.
5. Chung, J.-W.; Park, S.H.; Kim, N.; Kim, W.-J.; Park, J.H.; Ko, Y.; Yang, M.H.; Jang, M.S.; Han, M.-K.; Jung, C.; et al. Trial of ORG 10172 in Acute Stroke Treatment (TOAST) Classification and Vascular Territory of Ischemic Stroke Lesions Diagnosed by Diffusion-Weighted Imaging. *J. Am. Heart Assoc.* **2014**, *3*, doi:10.1161/JAHA.114.001119.
6. Marees, A.T.; de Kluiver, H.; Stringer, S.; Vorspan, F.; Curis, E.; Marie-Claire, C.; Derks, E.M. A Tutorial on Conducting Genome-Wide Association Studies: Quality Control and Statistical Analysis. *Int. J. Methods Psychiatr. Res.* **2018**, *27*, 1–10, doi:10.1002/mpr.1608.
7. Carrera, C.; Cullell, N.; Aguila, N.T.-; Bustamante, A.; Molina, C.A.; Giralt-steinhauer, E.; Mola-caminal, M.; Roquer, J.; Obach, V.; Arenillas, J.F.; et al. Validation of a Clinical-Genetics Score to Predict Hemorrhagic Transformations after RtPA. **2019**, 851–863, doi:10.1212/WNL.0000000000007997.
8. Roquer, J.; Rodríguez-Campello, A.; Gomis, M.; Jiménez-conde, J.; Cuadrado-godia, E.; Vivanco, R.; Giralt, E.; Sepúlveda, M.; Pont-Sunyer, C.; Cucurella, G.; et al. Acute Stroke Unit Care and Early Neurological Deterioration in Ischemic Stroke. *J. Neurol.* **2008**, *255*, 1012–7, doi:10.1007/s00415-008-0820-z.
9. Robberecht, W.; Vanhees, L.; Lemmens, R.; Pandolfo, M.; Thijs, V.; Goris, A. Variant on 9p21 Strongly Associates with Coronary Heart Disease , but Lacks Association with Common Stroke. **2009**, *4*, 1287–1293, doi:10.1038/ejhg.2009.42.
10. Heitsch, L.; Ibanez, L.; Carrera, C.; Pera, J.; Jimenez-Conde, J.; Slowik, A.; Strbian, D.; Fernandez-Cadenas, I.; Montaner, J.; Lee, J.-M. Meta-Analysis of Transethnic Association (MANTRA) Reveals Loci Associated With Neurological Instability After Acute Ischemic Stroke. In Proceedings of the International Stroke Conference; 2017.
11. Caty Carrera , Jara Cárcel-Márquez , Natalia Cullell, Nuria Torres-Águila, Elena Muiño , José Castillo , Tomás Sobrino, Francisco Campos , Emilio Rodríguez-Castro , Laia Llucia-Carol, Mònica Millán, Lucía Muñoz-Narbona , Elena López-Cancio , Alejandro Bus, I.F.-C. Single Nucleotide Variations in ZBTB46 Are Associated with Post-Thrombolytic Parenchymal Haematoma. *Brain* **2021**, doi:doi: 10.1093/brain/awab090.
12. Jiang, L.; Zheng, Z.; Qi, T.; Kemper, K.E.; Wray, N.R.; Visscher, P.M.; Yang, J. A Resource-Efficient Tool for Mixed Model Association Analysis of Large-Scale Data. *Nat. Genet.* **2019**, *51*, 1749–1755, doi:10.1038/s41588-019-0530-8.
